# A bioelectromagnetic hypothesis of chronic primary pain: from thalamocortical dysrhythmia to the consciousness-brain interface

**DOI:** 10.3389/fpain.2026.1790293

**Published:** 2026-06-04

**Authors:** Muhammad Khatib, Dror Robinson, Mustafa Yassin

**Affiliations:** 1 Orthopedic Research Unit, Hasharon Hospital, Rabin Medical Center, Petah Tikva, Israel; 2 Gray Faculty of Medicine, Tel Aviv University, Tel Aviv, Israel; 3 Department of Orthopedics, Rabin Medical Center, Petah Tikva, Israel

**Keywords:** bioelectromagnetic field, central sensitization, chronic pain, heart rate variability, neuroinflammation, thalamocortical dysrhythmia, ultra-weak photon emission

## Abstract

The World Health Organization's 2019 recognition in ICD-11 that chronic primary pain constitutes a disease in its own right demands novel conceptual frameworks. Current models focusing on peripheral and central sensitization, while valuable, may describe downstream manifestations rather than primary etiology. We hypothesize that chronic pain may arise from disruption of bioelectromagnetic coherence at the interface where consciousness and neural tissue interact—upstream of the cytokine cascades, neuroinflammation, central sensitization, and epigenetic modifications typically studied as pain mechanisms. Convergent lines of evidence support this hypothesis: (1) thalamocortical dysrhythmia documented in chronic pain patients via magnetoencephalography, with therapeutic correction producing pain relief; (2) heart rate variability abnormalities and reduced cardiac coherence consistently found in chronic pain populations; (3) photobiomodulation efficacy in randomized controlled trials suggesting electromagnetic etiology; (4) mitochondrial bioenergetic dysfunction preceding inflammatory cascades; (5) ultra-weak photon emission alterations correlating with disease states; and (6) circadian rhythm disruption patterns in chronic pain conditions. This framework positions inflammatory cascades, glial activation, and central sensitization as potential downstream consequences of bioelectromagnetic disruption rather than primary causes. The hypothesis generates falsifiable predictions and suggests novel therapeutic approaches targeting electromagnetic coherence restoration.

## 1 Introduction

Chronic pain affects approximately 20% of the global adult population, representing one of the leading causes of disability worldwide and imposing an annual economic burden exceeding $600 billion in the United States alone (1–3). Despite intensive research spanning decades, current pharmacological and interventional approaches provide inadequate relief for the majority of patients, with opioid therapies contributing to a parallel crisis of addiction and mortality (4).

The 2019 adoption by the World Health Organization (WHO) of the International Classification of Diseases 11th Revision (ICD-11) marked a paradigm shift in pain medicine (5–7). For the first time, the classification system includes a systematic taxonomy for chronic pain conditions developed by the International Association for the Study of Pain (IASP) Task Force. Most significantly, ICD-11 introduced the novel category of chronic primary pain—defined as pain persisting for three months or more with significant emotional distress and/or functional disability, conceived as a disease in its own right rather than merely a symptom of underlying pathology (5). This category encompasses conditions previously considered diagnostically orphaned, including fibromyalgia, chronic widespread pain, and nonspecific low back pain.

An important terminological clarification is necessary before proceeding. Throughout this paper, the term *bioelectromagnetic coherence* refers to the temporal and spatial coordination of electromagnetic field oscillations generated by synchronized neural activity, as measurable by MEG, EEG, and MCG—following the framework established by McFadden's Conscious Electromagnetic Information (CEMI) Field Theory (8), in which the brain's endogenous EM field is understood as a classical macroscopic structure arising from coordinated neuronal firing. Quantum coherence in the strict decoherence physics sense is not invoked. Disruption of this coordination—whether through altered oscillatory frequency, reduced inter-regional synchrony, or abnormal spectral power distribution—constitutes the “bioelectromagnetic incoherence” proposed to underlie chronic primary pain.

A further distinction is essential to situate this hypothesis correctly. This framework does *not* propose an alternative account of acute pain, whose nociceptive basis—peripheral receptor activation, action potential propagation, and central relay—is well-established and not in question here. Our hypothesis addresses exclusively the transition from acute to chronic pain and the maintenance of chronic primary pain in the absence of ongoing tissue pathology. The central clinical puzzle we address is why, after peripheral healing, pain persists and chronifies in some patients but resolves in others. Growing evidence indicates that chronification is predicted by central, not peripheral, features: pre-morbid thalamocortical connectivity patterns (9), oscillatory abnormalities (10), and autonomic dysregulation (11)—all of which are upstream of the molecular sensitization cascades typically targeted by pharmacotherapy.

This reconceptualization demands new theoretical frameworks. If chronic primary pain is indeed a disease entity rather than a symptom, what is its etiology? Current dominant models—peripheral sensitization, central sensitization, neuroinflammation, and maladaptive neuroplasticity—provide mechanistic descriptions of how pain signals are amplified and perpetuated, but they do not fully address why the system becomes dysregulated in the first place, nor do they account for the subjective, conscious experience of suffering that defines pain as a phenomenon (12–14).

We propose here a bioelectromagnetic field hypothesis of chronic pain that addresses these gaps by situating the primary pathology at the interface where consciousness and neural tissue interact. This framework draws upon converging evidence from thalamocortical dysrhythmia research, heart rate variability studies, biophotonics, and photobiomodulation trials to suggest that chronic pain may represent a disruption in the electromagnetic coherence through which consciousness interfaces with the body—a disruption that may be upstream of the molecular and cellular cascades currently studied as pain mechanisms.

## 2 Theoretical foundations: the brain as receiver

### 2.1 The transmissive theory of brain function and CEMI field theory

The dominant paradigm in neuroscience—what William James termed the “productive” theory—holds that consciousness is generated entirely by neural activity, an epiphenomenon of electrochemical processes in the brain (15). However, James himself articulated an alternative: the “transmissive” or “filter” theory, proposing that the brain's function may be “permissive or transmissive” rather than solely productive (16). In his 1898 Ingersoll Lecture, James stated: “My thesis is now this: that, when we think of the law that thought is a function of the brain, we are not required to think of productive function only; we are entitled also to consider permissive or transmissive function”.

James employed the analogy of a prism, which does not create the spectrum of colors from white light but rather filters and separates wavelengths that exist independently of the prism itself (15, 16). The brain, in this view, may similarly filter, channel, and focus a broader field of consciousness rather than generating it *de novo*. This perspective was elaborated by Henri Bergson, who argued that the brain serves to select and narrow consciousness for practical, survival-related purposes, and by Aldous Huxley, who popularized the concept of the brain as a “reducing valve” (17, 18).

It should be noted that the transmissive theory remains a minority position within mainstream neuroscience, which generally favors the productive model. Nevertheless, recent scholarship has revisited this framework in light of contemporary findings (15). Rouleau and colleagues, reviewing evidence that the brain receives, processes, and emits electromagnetic fields and ultraweak photon emissions, argue that if any subset of brain function is explained by causal elements outside the head that impinge directly upon neural tissues, brain function cannot be fully explained by a purely productive model. The discovery of ephaptic coupling, endogenous biophoton signaling, and sensitivity to environmental electromagnetic fields provides candidate mechanisms for transmissive brain function (15, 19).

A complementary and independently developed framework is McFadden's Conscious Electromagnetic Information (CEMI) Field Theory (8). The CEMI framework proposes that consciousness is associated with the brain's endogenous classical electromagnetic field, which both integrates information from the synchronized firing of neuronal populations and exerts downward causal influence on action potential generation via electromagnetic induction. This framework is empirically grounded: the brain's EM field is measurable by MEG and EEG, and its properties—coherence, synchrony, spectral composition—correlate with conscious states. Within the CEMI framework, disruption of field coherence has direct experiential consequences, providing a mechanism through which thalamocortical dysrhythmia translates into persistent subjective pain. The CEMI theory thus provides mechanistic scaffolding for the consciousness-brain interface disruption central to our hypothesis, without invoking quantum or metaphysical constructs. Convergently, Ambron (20) demonstrated that the persistence and subjectivity of pain in the anterior cingulate cortex is maintained via synchronized oscillating electromagnetic waves, with synaptic sensitization sustaining the field oscillations that constitute the conscious pain experience. This independent line of evidence provides direct empirical support for the proposition that chronic pain at the conscious level is an electromagnetic field phenomenon.

That consciousness depends on the spatiotemporal organization of cortical electromagnetic activity—not merely its presence—is further supported by anesthesia research. Bhattacharya et al. (21) demonstrated that propofol-induced loss of consciousness reorganizes cortical traveling waves in non-human primates: slow-delta waves become more spatially coherent, more stereotyped, and dominant, displacing higher-frequency (8–30 Hz) waves associated with cognition into alternative directional channels. Consciousness is lost not because electromagnetic activity ceases, but because its functional organization is disrupted. Liang et al. (22), using stereo-EEG in thalamocortical and cortico-cortical systems, showed that propofol-induced unconsciousness reduces cortical complexity while functional connectivity increases or remains stable, with a directional shift in information flow such that the prefrontal cortex and thalamus begin driving temporal lobe activity rather than maintaining balanced bidirectional exchange. Convergently, Choe et al. (23) confirmed in a large intracranial EEG cohort (*n* = 73) that propofol-induced unconsciousness increases global functional connectivity while reducing network complexity and efficiency, with posterior connectivity emerging as the most informative predictor of conscious state. These findings directly implicate the organization of thalamocortical electromagnetic dynamics—not the quantity of neural activity—as a substrate of conscious states, consistent with the CEMI framework and with the hypothesis that chronic pain represents a specific pattern of electromagnetic disorganization at this interface.

### 2.2 Implications for pain: consciousness as primary

Pain, by definition, is a conscious experience. The International Association for the Study of Pain defines pain as “an unpleasant sensory and emotional experience associated with, or resembling that associated with, actual or potential tissue damage” (24). Crucially, pain requires consciousness—it cannot exist without a conscious subject to experience it. This suggests that any complete theory of chronic pain must account for the role of consciousness, not merely as an emergent property of nociceptive processing, but as fundamental to the phenomenon.

If the brain functions, at least in part, as a receiver or filter of consciousness—and if consciousness interfaces with neural tissue through electromagnetic mechanisms—then chronic pain may represent a disturbance at this interface. Rather than being solely a matter of aberrant neural signaling or inflammatory cascades, chronic pain may fundamentally involve disruption in the bioelectromagnetic coherence through which consciousness connects with the body. This perspective suggests a different causal hierarchy than the conventional view: rather than nociceptive signals causing conscious pain experience, disruption at the consciousness-brain interface may generate the conditions for chronic pain to emerge and persist.

## 3 The upstream hypothesis: bioelectromagnetic disruption as proposed primary etiology

### 3.1 Causal architecture: From field disruption to molecular cascade

**Figure 1:**
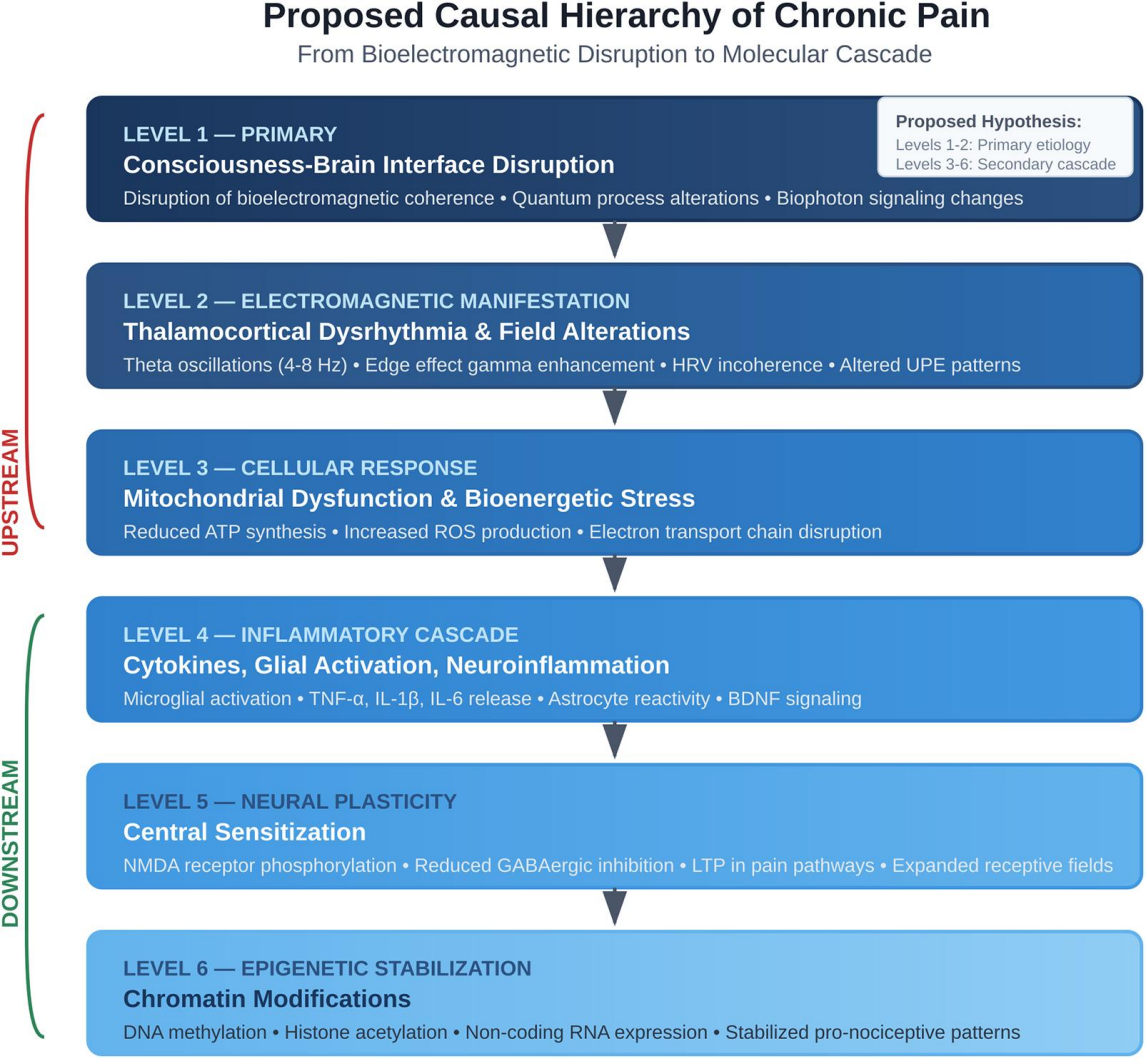
Proposed causal hierarchy of chronic pain. The framework positions bioelectromagnetic disruption at the consciousness-brain interface (Level 1) as primary etiology, with thalamocortical dysrhythmia (Level 2), mitochondrial dysfunction (Level 3), neuroinflammation (Level 4), central sensitization (Level 5), and epigenetic modifications (Level 6) as downstream consequences. Current research focuses on Levels 4–6; this hypothesis suggests Levels 1–2 may be causally prior. TCD, thalamocortical dysrhythmia; ROS, reactive oxygen species; ETC, electron transport chain; BDNF, brain-derived neurotrophic factor; TNF-α, tumor necrosis factor-alpha; IL, interleukin; NMDA, N-methyl-D-aspartate.

We propose a hypothesized causal chain linking bioelectromagnetic field disruption to the established mechanisms of chronic pain (Figure 1):

#### 3.1.1 Level 1 (primary): consciousness-brain interface disruption

We hypothesize that chronic pain may originate from disturbance in the bioelectromagnetic coherence through which consciousness interfaces with neural tissue. Within the CEMI framework, this disruption alters the properties of the brain's endogenous EM field, producing experiential consequences directly. This may additionally involve altered biophoton signaling within neurons, or disturbance of the electromagnetic fields generated by coherent neural oscillations. The subjective experience of chronic pain—its “felt” quality—would exist at this level.

#### 3.1.2 Level 2 (electromagnetic manifestation): thalamocortical dysrhythmia and field alterations

The consciousness-level disruption may manifest electromagnetically as thalamocortical dysrhythmia (TCD)—characterized by persistent low-frequency oscillations (4–8 Hz theta activity) in thalamocortical circuits and edge effect phenomena in cortical areas adjacent to abnormally oscillating regions (10, 25). Altered biophoton emission patterns and disrupted heart rhythm coherence also manifest at this level.

#### 3.1.3 Level 3 (cellular response): mitochondrial dysfunction and bioenergetic stress

Electromagnetic incoherence at the cellular level may manifest as mitochondrial dysfunction—reduced ATP synthesis, increased reactive oxygen species production, and disrupted electron transport chain function (26–28). Mitochondrial dysfunction precedes and potentiates inflammatory responses.

#### 3.1.4 Level 4 (inflammatory cascade): cytokines, glial activation, neuroinflammation

Mitochondrial dysfunction and cellular stress activate innate immune responses. Microglia shift to pro-inflammatory phenotypes, releasing cytokines (TNF-α, IL-1β, IL-6), chemokines, and brain-derived neurotrophic factor (29, 30). These processes are well-documented in chronic pain but, in our framework, may represent secondary responses to upstream bioelectromagnetic disruption.

#### 3.1.5 Level 5 (neural plasticity): central sensitization

Sustained neuroinflammation drives maladaptive synaptic plasticity—long-term potentiation in pain pathways, reduced inhibitory neurotransmission, NMDA receptor phosphorylation, and expanded receptive fields (31, 32). Central sensitization emerges as a downstream adaptation. Synaptic plasticity in the pain-related cingulate and insular cortex (33) represents an important perpetuating mechanism within this framework—acknowledged as downstream rather than primary.

#### 3.1.6 Level 6 (epigenetic stabilization): chromatin modifications

Persistent signaling drives epigenetic changes—DNA methylation, histone acetylation, and non-coding RNA expression—that stabilize pro-nociceptive gene expression patterns (34, 35). These modifications contribute to chronicity but may represent the most downstream level of the cascade.

### 3.2 Resolution of the specificity problem

A critical question is why electromagnetic disruption manifests as chronic pain in some cases but as other conditions (tinnitus, Parkinson's disease, depression) in others, given that thalamocortical dysrhythmia is documented across these conditions (25). We propose that specificity arises from three factors:

#### 3.2.1 Anatomical localization

TCD affecting sensory thalamocortical circuits manifests as chronic pain, while TCD in auditory circuits produces tinnitus, and TCD in motor circuits contributes to Parkinson's symptoms. The location of disruption determines symptom nature.

#### 3.2.2 Pattern and frequency characteristics

The specific oscillatory patterns, phase relationships, and coherence characteristics differ across conditions. Chronic pain shows distinctive cross-frequency coupling and altered gamma oscillations in pain-processing regions (36).

#### 3.2.3 Individual vulnerability factors

Genetic polymorphisms, prior sensitization, psychological factors, and developmental history create individual-specific vulnerabilities that channel bioelectromagnetic disruption toward particular phenotypic expressions (37, 38).

Notably, comorbidity between TCD conditions is the rule rather than the exception—chronic pain patients frequently experience depression, anxiety, tinnitus, and cognitive dysfunction—supporting the concept of a shared upstream mechanism with variable downstream manifestations.

Crucially, the anatomical localization argument is not merely definitional. Each thalamic nucleus occupies a distinct position within the thalamocortical connectome and subserves a specific functional domain: the ventral posterolateral and posteromedial nuclei (VPL/VPM) relay somatosensory and nociceptive information to S1/S2 and insular cortex; the medial geniculate nucleus relays auditory information to temporal cortex; the ventral lateral and ventral anterior nuclei relay motor signals from basal ganglia and cerebellum to premotor and motor cortex. Dysrhythmia in each of these circuits therefore disrupts coherence within a functionally distinct information-processing stream, producing domain-specific experiential consequences—not because “pain circuits cause pain” as a tautology, but because the representational content carried by each thalamocortical loop determines the phenomenological character of its disruption. This principle is directly supported by the prospective findings of Cannistra, Saccà et al. (9), who demonstrated that reduced functional connectivity specifically between VPL thalamus and left dorsolateral prefrontal cortex—a somatosensory-prefrontal circuit, not a generic thalamocortical pathway—predicted pain chronification with AUC = 0.79, the only cluster surviving whole-brain correction. Had the chronification-predictive signal resided in auditory or motor thalamocortical connectivity, the hypothesis would face a serious challenge; instead, the anatomical specificity of the finding validates the circuit-dependent model of TCD phenotypes. Llinás and colleagues themselves characterized TCD as a unitary dysrhythmic syndrome whose clinical expression is determined by which thalamocortical columns are affected (25), a formulation that treats circuit topology as the determinant of phenotype rather than invoking separate mechanisms for each condition.

### 3.3 Acknowledgment of bidirectional processes

**Figure 2:**
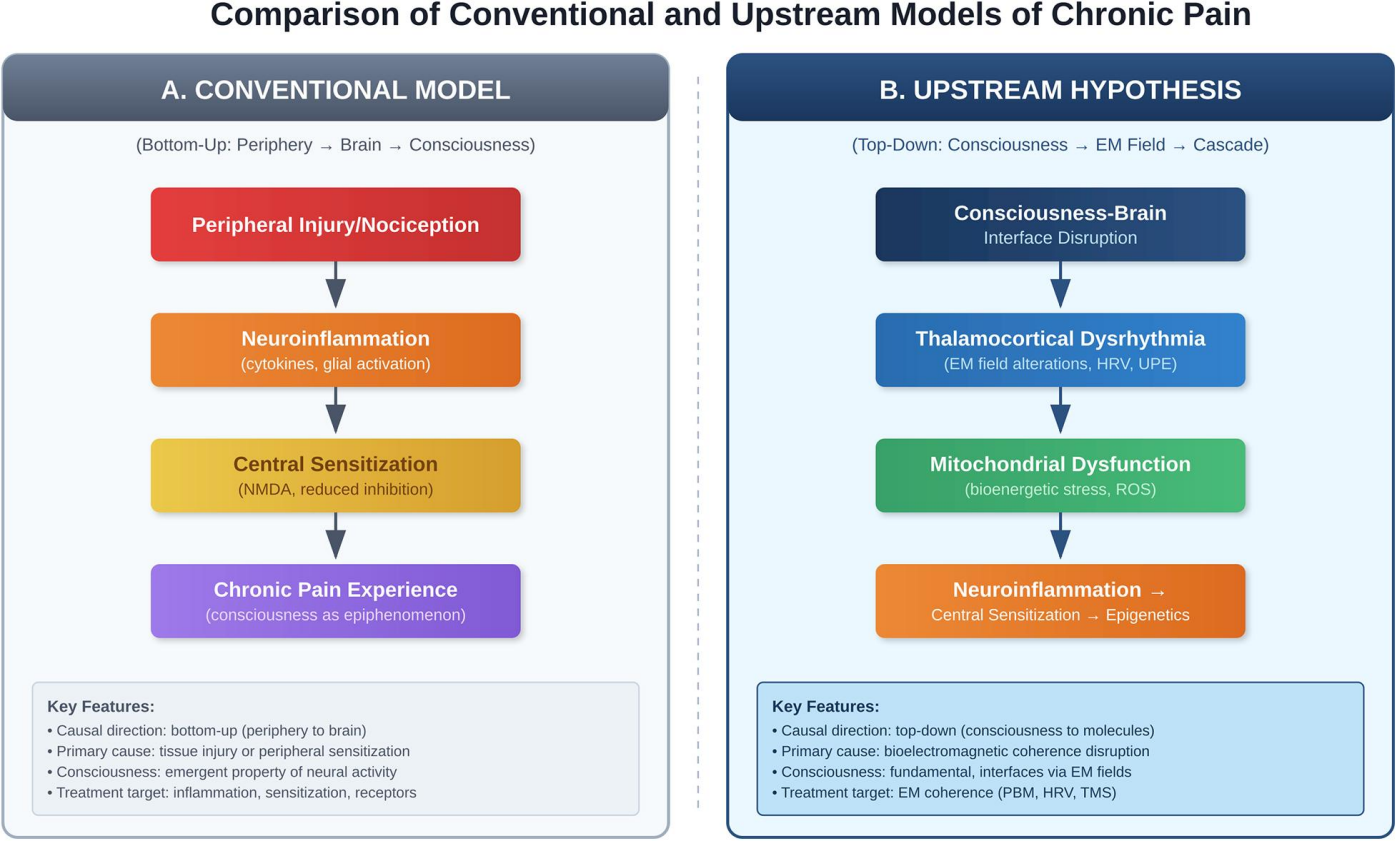
Comparison of conventional and upstream models of chronic pain. **(A)** The conventional bottom-up model posits that peripheral injury or nociception drives neuroinflammation and central sensitization, with conscious pain as an emergent epiphenomenon of neural activity. Treatment targets in this model are primarily peripheral receptors, inflammatory mediators, and sensitization mechanisms (Levels 4–6). **(B)** The upstream bioelectromagnetic hypothesis proposes top-down causation: primary disruption at the consciousness-brain interface cascades through thalamocortical dysrhythmia, mitochondrial dysfunction, neuroinflammation, and central sensitization. In this framework, Levels 4–6 are downstream consequences rather than primary causes, and treatment targets include electromagnetic coherence restoration (photobiomodulation, HRV biofeedback, TMS, DBS). Key features: causal direction, top-down (consciousness to molecules); primary cause, bioelectromagnetic coherence disruption; treatment target, electromagnetic coherence (PBM, HRV, TMS/DBS).

While this hypothesis emphasizes top-down causation from consciousness-brain interface disruption to downstream molecular cascades, bidirectional and parallel processes likely operate in chronic pain. Inflammatory mediators can feed back to alter thalamic and cortical oscillatory dynamics; peripheral nociceptive input can drive central changes; and epigenetic modifications may influence electromagnetic properties of neural tissue. The upstream framework does not deny these reciprocal influences but proposes that the initiating disturbance—the “first domino”—may often reside at the bioelectromagnetic level rather than the molecular level (Figure 2).

## 4 Integration with established pain neuroscience

### 4.1 Central sensitization: A downstream consequence

Central sensitization—defined as increased responsiveness of nociceptive neurons in the central nervous system to normal or subthreshold afferent input—is a well-validated mechanism in chronic pain (31, 32). The phenomenon involves phosphorylation of NMDA receptors, reduced GABAergic and glycinergic inhibition, altered descending modulation, and structural synaptic reorganization.

In the upstream framework, central sensitization is proposed as not the cause of chronic pain but rather one of its most important perpetuating mechanisms—a downstream adaptation occurring in response to sustained bioelectromagnetic disruption. Evidence consistent with this position includes: (1) central sensitization takes time to develop, whereas TCD can be present early in pain chronification (39); (2) interventions targeting electromagnetic coherence can reduce pain even when central sensitization biomarkers remain present (40, 41); (3) the correlation between central sensitization degree and subjective pain intensity is imperfect (42).

### 4.2 Neuroinflammation and glial activation

Microglial activation and astrocyte reactivity are recognized as critical contributors to chronic pain pathophysiology (29, 30, 43). Activated microglia release pro-inflammatory cytokines, chemokines, and neuromodulators that enhance nociceptive transmission. We position this neuroinflammatory cascade as potentially secondary to upstream bioelectromagnetic disruption. Supporting evidence includes: (1) mitochondrial dysfunction activates innate immunity and inflammasome assembly (44, 45); (2) inflammatory cytokines show circadian variation linked to the suprachiasmatic nucleus (46); (3) anti-inflammatory interventions often fail to resolve chronic pain (47).

### 4.3 Sex differences: differential electromagnetic vulnerability

Chronic pain affects women at rates 2–3 times higher than men, with over 50% of chronic pain conditions more prevalent in women (48–50). The bioelectromagnetic framework suggests additional mechanisms. Estrogen and progesterone have documented effects on neural excitability, ion channel function, and mitochondrial activity—all factors influencing bioelectromagnetic coherence (51, 52). Fluctuating hormone levels across the menstrual cycle, pregnancy, and menopause would create varying states of electromagnetic vulnerability.

### 4.4 Genetics and epigenetics: modulators of vulnerability

Substantial evidence supports genetic contributions to chronic pain susceptibility. Polymorphisms in genes encoding ion channels (SCN9A, SCN10A, SCN11A), catecholamine metabolism (COMT), opioid receptors (OPRM1), and inflammatory mediators influence pain sensitivity and chronification risk (37, 38, 53). Within the upstream framework, genetic factors would not cause chronic pain directly but rather modulate vulnerability to bioelectromagnetic disruption. A genetic variant affecting sodium channel kinetics may alter electromagnetic properties of neuronal membranes, making certain circuits more susceptible to coherence disruption.

Epigenetic modifications—DNA methylation, histone acetylation, and non-coding RNA expression—are extensively documented in chronic pain (34, 35). We position these as potentially the most downstream level—representing stabilization of changes initially triggered by bioelectromagnetic disruption.

### 4.5 Psychological dimensions: consciousness-level factors

Psychological factors—pain catastrophizing, fear-avoidance, depression, anxiety—are among the strongest predictors of chronic pain development and maintenance (54–56). The bioelectromagnetic framework offers a different perspective: psychological factors may operate at the consciousness level where pain originates. Pain catastrophizing is associated with enhanced brain responses to nociceptive stimuli and reduced conditioned pain modulation—if chronic pain originates at the consciousness-brain interface, catastrophizing may directly amplify the disturbance by focusing conscious attention on the pain experience.

The strong comorbidity between chronic pain and depression/anxiety may reflect shared bioelectromagnetic pathophysiology rather than purely psychological relationships (57). All three conditions involve disrupted neural oscillatory dynamics, reduced HRV coherence, and altered inflammatory markers—consistent with a common upstream disturbance manifesting in different symptomatic expressions.

### 4.6 Developmental and lifespan considerations

Chronic pain onset patterns differ across the lifespan, with peaks in adolescence/young adulthood and again in later life (58). Adverse childhood experiences (ACEs) substantially increase risk of adult chronic pain, with dose-response relationships documented between ACE scores and pain prevalence (59, 60). These observations require explanation within any comprehensive pain theory.

From the bioelectromagnetic perspective, early life represents a critical period for development of the consciousness-brain interface. The maturation of thalamocortical circuits, myelination of long-range connections, and development of coherent oscillatory dynamics all occur during childhood and adolescence (61). Adverse experiences during this period—particularly chronic stress, trauma, or neglect—may disrupt the normal development of bioelectromagnetic coherence, creating lifelong vulnerability. The stress-induced alterations in hypothalamic-pituitary-adrenal axis function documented in ACE survivors would affect circadian rhythms, autonomic regulation, and inflammatory responses—all factors that influence bioelectromagnetic coherence (62).

The increased prevalence of chronic pain in older adults may reflect cumulative degradation of bioelectromagnetic coherence mechanisms. Mitochondrial dysfunction accumulates with age, circadian amplitude diminishes, sleep architecture changes, and neurodegenerative processes affect thalamocortical circuit integrity (63, 64). The “inflammaging” phenomenon—chronic low-grade inflammation associated with aging—may represent downstream manifestation of progressive bioelectromagnetic incoherence rather than a primary driver of age-related pain.

## 5 Empirical evidence for the bioelectromagnetic framework

**Figure 3:**
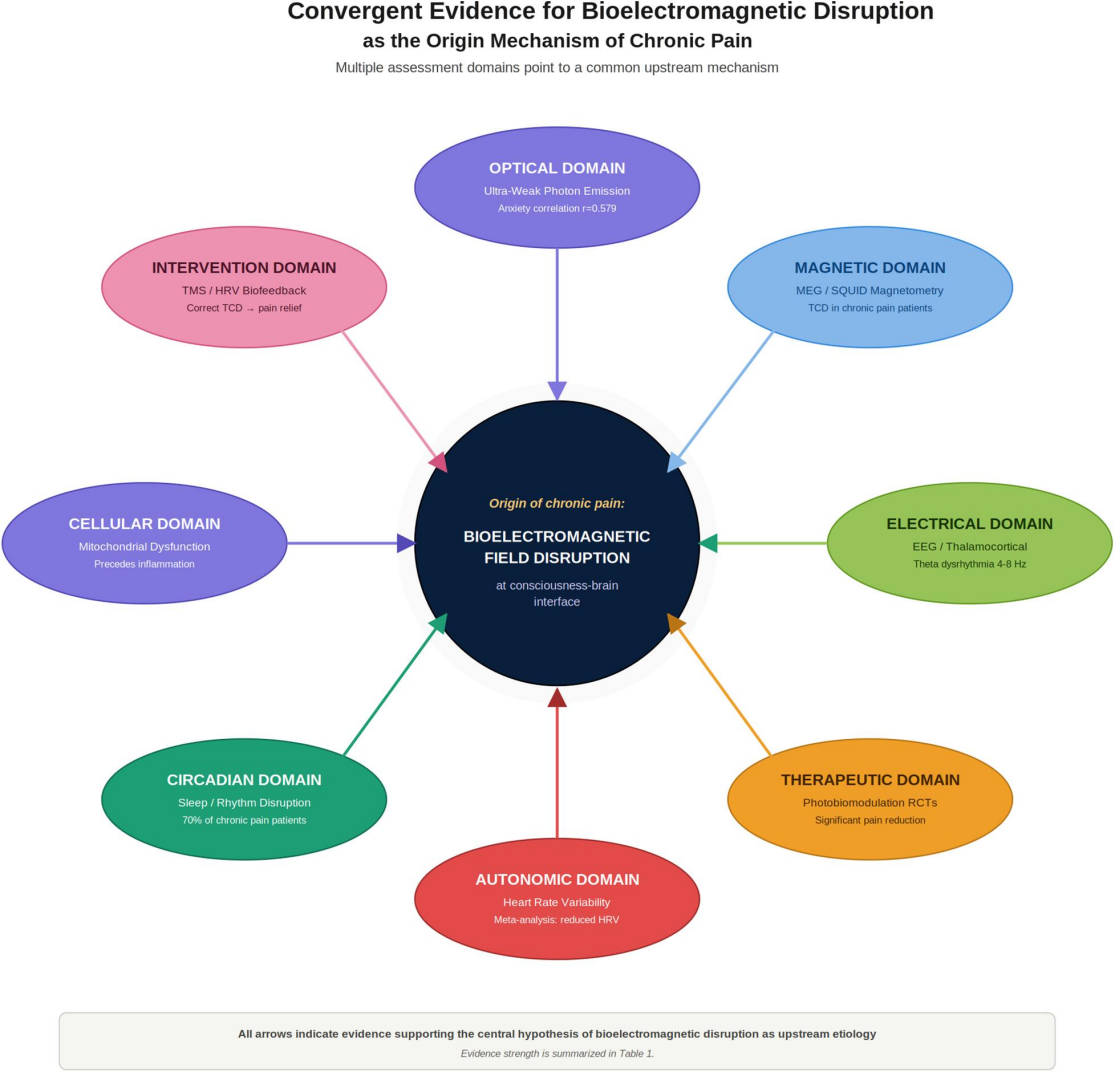
Convergent evidence for bioelectromagnetic disruption in chronic pain. Eight independent evidence domains—optical (ultra-weak photon emission, UPE), magnetic (magnetoencephalography, MEG), electrical (electroencephalography, EEG; thalamocortical dysrhythmia, TCD), therapeutic (photobiomodulation, PBM), autonomic (heart rate variability, HRV), circadian rhythm, cellular (mitochondrial dysfunction), and interventional (transcranial magnetic stimulation, TMS; deep brain stimulation, DBS; HRV biofeedback)—converge on bioelectromagnetic field disruption as a common upstream mechanism in chronic primary pain. All arrows indicate supporting evidence for the central hypothesis. Evidence strength is summarized in Table 1.

Multiple independent lines of evidence converge to support the hypothesis that bioelectromagnetic disruption may underlie chronic pain pathophysiology (Figure 3, Table 1).

**Table 1:** Summary of evidence supporting the bioelectromagnetic hypothesis of chronic pain.

Evidence Domain	Key Findings	Key References	Evidence Strength
Thalamocortical Dysrhythmia	Increased theta (4–8 Hz) power in thalamus/cortex with edge effect gamma enhancement; TCD predicts chronification (AUC 0.79); DBS/TMS correction produces pain relief	(9, 10, 25, 65)	Strong (MEG/EEG + therapeutic + prospective)
Heart Rate Variability	Reduced HRV and parasympathetic dysfunction consistent across multiple chronic pain conditions	(11) (meta-analysis)	Strong (meta-analytic)
Photobiomodulation	Significant pain reduction in RCTs for low back pain, neck pain, TMD, neuropathic pain	(66, 67)	Strong (RCTs, systematic reviews)
Ultra-Weak Photon Emission	UPE alterations in disease states; *r* = 0.579 with anxiety vs. *r* = 0.093 with pain in chronic pain cohort	(68, 69)	Moderate (emerging pain-specific data)
Mitochondrial Dysfunction	Reduced ATP synthesis, increased ROS, ETC disruption; precedes and potentiates inflammation	(26, 45)	Strong (mechanistic)
Circadian Disruption	70% of chronic pain patients have sleep disorders vs. 17% general population; melatonin rhythm disruption	(70, 71)	Strong (epidemiological)
Consciousness-EM Interface	Synchronized oscillating EM waves sustain conscious pain in ACC; CEMI field disruption has direct experiential consequences	(8, 20)	Moderate–Strong (direct evidence)

### 5.1 Thalamocortical dysrhythmia: the central evidence

Magnetoencephalography (MEG) studies have documented thalamocortical dysrhythmia (TCD) in chronic pain patients, characterized by increased low-frequency (theta, 4–8 Hz) oscillatory power in thalamus and cortex, with “edge effect” gamma-frequency enhancement in adjacent cortical regions (10, 25, 72). Llinás and colleagues proposed that TCD results from hyperpolarization of thalamic relay neurons, shifting them into low-frequency bursting modes that disrupt normal thalamocortical information processing. TCD has been most consistently documented in neuropathic pain and fibromyalgia; prevalence across other pain phenotypes requires further study.

Critically, therapeutic interventions that correct TCD also relieve pain. Thalamotomy and deep brain stimulation (DBS) that normalize thalamic oscillatory patterns produce pain relief (10). Fontaine et al. (73) demonstrated that DBS of the anterior cingulate cortex combined with sensory thalamic stimulation in refractory chronic neuropathic pain was safe and produced significant quality-of-life improvement (EQ-5D utility score, *p* = 0.039 during active ACC stimulation; *p* = 0.034 at end of study), while pain intensity did not change significantly—suggesting that electromagnetic modulation of the affective pain-processing circuit at the cingulate level operates independently of peripheral nociceptive transmission, consistent with an upstream mechanism. Transcranial magnetic stimulation (TMS) targeting dysrhythmic cortical regions also shows robust efficacy in chronic pain (74). De Martino et al. (75), using TMS-EEG to probe evoked oscillatory dynamics during experimentally induced acute pain, demonstrated that pain reduces *α*-band power locally at M1 and disrupts *α*-band phase synchronization in remote parietal–occipital regions, with the magnitude of disruption correlating with individual pain thresholds (cold: rho = 0.638, *p* = 0.001; heat: rho = −0.463, *p* = 0.023)—providing direct electrophysiological evidence that pain states alter electromagnetic coherence across thalamocortical networks in a frequency-specific and circuit-dependent manner. These converging therapeutic and electrophysiological observations provide strong evidence for a causal role of electromagnetic dysrhythmia in chronic pain.

The relationship between brain rhythms and pain has been comprehensively characterized by Ploner, Sorg and Gross (65), who demonstrated that pain involves dynamic, multi-frequency interactions across the salience network, default-mode network, and sensorimotor cortex, with alpha desynchronization, gamma enhancement, and theta entrainment characterizing the acute pain response. Critically, in chronic pain states these normal rhythm dynamics are replaced by persistently altered oscillatory configurations—particularly tonic alpha suppression and elevated low-frequency power in thalamocortical projection areas—patterns entirely consistent with the TCD model. The Ploner et al. review thus contextualizes TCD within a broader brain rhythms framework for pain, strengthening the scientific foundation of the electromagnetic hypothesis.

The most direct prospective evidence linking thalamocortical connectivity specifically to pain chronification was recently provided by Cannistra, Saccà et al. (9). Using resting-state fMRI data from the UK Biobank and the independent OpenPain dataset, the study analyzed participants with acute musculoskeletal pain (*n* = 160) and categorized them based on recovery (AMPR) vs. development of chronic pain (CMPO). Thalamocortical and corticostriatal connectivity patterns predicted chronification with AUC values of 0.74–0.83 across cross-validation cohorts. Critically, participants who recovered (AMPR) showed significantly greater functional connectivity between the ventral posterolateral thalamus (VPL-Thal) and left DLPFC compared to those who chronified (CMPO)—meaning reduced VPL–DLPFC thalamocortical connectivity prospectively predicted pain chronification, with the VPL-Thal–left DLPFC cluster being the only finding to survive whole-brain correction (Cohen's d = 0.837, pFDR < 0.05, AUC = 0.79). Additionally, increased right NAc–mPFC corticostriatal connectivity characterized the CMPO group, consistent with reward-circuit involvement in chronification. These findings provide the first prospective neuroimaging evidence that disrupted thalamocortical connectivity—the anatomical substrate of TCD—predicts pain chronification, and directly support our proposed Experiment 1.

### 5.2 Heart rate variability and cardiac coherence

The heart generates the body's largest rhythmic electromagnetic field, approximately 100 times stronger than the brain's field and detectable several feet from the body using SQUID-based magnetometers (76, 77). Heart rate variability (HRV) reflects the dynamic interplay between sympathetic and parasympathetic activity and has emerged as an important biomarker in chronic pain.

A comprehensive meta-analysis by Tracy and colleagues found consistent evidence of reduced HRV and parasympathetic dysfunction across multiple pain conditions (11). The neurovisceral integration model links HRV to prefrontal cortical function and emotional regulation (78). HRV biofeedback interventions have shown efficacy in chronic pain management across multiple randomized controlled trials (41).

### 5.3 Photobiomodulation: therapeutic electromagnetic intervention

Photobiomodulation (PBM) involves application of red or near-infrared light (600–1,100 nm) for therapeutic benefit (67, 79). The primary mechanism involves absorption by cytochrome c oxidase in mitochondria, enhancing electron transport chain function, ATP synthesis, and reducing oxidative stress. Systematic reviews support PBM efficacy for low back pain, neck pain, temporomandibular disorders, and neuropathic pain (66, 67). Evidence quality varies across conditions and results are sensitive to treatment parameters. The efficacy of PBM is consistent with the electromagnetic framework: if electromagnetic coherence is primary to chronic pain, therapeutic electromagnetic interventions should provide relief.

### 5.4 Ultra-Weak photon emission research

Ultra-weak photon emission (UPE), also termed biophoton emission, refers to spontaneous photon emission from living systems (68, 80, 81). UPE originates primarily from oxidative metabolic processes. UPE alterations have been documented in multiple disease states including cancer, diabetes, and Alzheimer's disease (68, 82, 83).

Our research demonstrates differential validity of fingertip biophoton emission in chronic pain patients: UPE shows strong correlation with anxiety (*r* = 0.579) but negligible correlation with pain intensity (*r* = 0.093), a 6.2-fold difference (69). We initially interpreted this finding as evidence that UPE indexes a global state of bioelectromagnetic coherence common to both pain and anxiety. On reflection, that framing claims more than the data support: a measure genuinely reflecting global coherence would not be expected to show such strong specificity for a single affective dimension. A more constrained interpretation is that fingertip UPE preferentially samples the peripheral output of the autonomic–mitochondrial limb of the system, which is more tightly coupled to sympathetic and HPA-axis activity (and therefore to anxiety) than to thalamocortical pain processing. Within this revised view, UPE remains consistent with the bioelectromagnetic framework but functions as a partial, peripherally weighted biomarker rather than a comprehensive readout. Validation against additional measurement sites (e.g., scalp or peri-cardiac UPE) and across chronic pain phenotypes will be required before stronger claims about its scope are warranted.

### 5.5 Sleep and circadian rhythms

Sleep disturbance and chronic pain share a bidirectional relationship (70, 84, 85). Up to 70% of chronic pain patients report sleep disorders vs. approximately 17% of the general population. Circadian rhythms are fundamentally electromagnetic phenomena, with the master clock in the suprachiasmatic nucleus generating rhythmic neural oscillations (86). Melatonin has documented analgesic properties, and disruption of melatonin secretion rhythm is documented in fibromyalgia (71, 87).

The upstream framework suggests that circadian disruption may impair the bioelectromagnetic coherence necessary for consciousness-brain interface function. Sleep, particularly slow-wave sleep, appears to serve restorative functions for neural oscillatory dynamics and may be necessary for “resetting” electromagnetic coherence (88).

Critically, the directionality of the sleep–pain relationship is not solely from pain to sleep disruption. Prospective cohort studies demonstrate that sleep disturbance precedes and predicts the onset of chronic pain. In the Norwegian HUNT cohort, Mork and Nilsen (89) showed that women reporting sleep problems at baseline had a markedly elevated risk of developing fibromyalgia over 10-year follow-up, with a dose-response relationship between severity of sleep complaints and incident disease. Generaal et al. (90), in a 6-year prospective analysis of the Netherlands Study of Depression and Anxiety, found that insomnia symptoms and short sleep duration predicted the onset of chronic multisite musculoskeletal pain. Finan, Goodin, and Smith (70), reviewing the prospective evidence then available, concluded that sleep disturbance is a more reliable predictor of subsequent pain than pain is of subsequent sleep disturbance. Within the bioelectromagnetic framework, this temporal ordering is informative: persistent disruption of the oscillatory machinery that depends on intact sleep architecture would be expected to precede the chronification of pain rather than follow it, consistent with sleep loss acting at the level of electromagnetic coherence restoration rather than as a mere consequence of nociceptive input.

A second observation deserves attention: a number of sleep aids beyond melatonin have established analgesic effects in chronic pain conditions, and these effects appear to be selective by mechanism rather than by hypnotic potency. Low-dose tricyclic antidepressants (amitriptyline, nortriptyline) remain first-line agents for fibromyalgia and several neuropathic pain conditions, with meta-analytic evidence of moderate effects on both pain and sleep disturbance in fibromyalgia (91). Bedtime very-low-dose cyclobenzaprine improves both pain and sleep physiology, with parallel reductions in pain and improvements in non-REM sleep architecture in fibromyalgia (92). The gabapentinoid pregabalin increases slow-wave sleep and reduces pain in fibromyalgia, with polysomnographic confirmation that the increase in stage N3 sleep parallels the analgesic effect (93, 94). Conversely, the benzodiazepine-receptor agonist zolpidem improves subjective sleep in fibromyalgia but, in controlled study, does not reduce pain (95)—a dissociation that is informative because zolpidem produces sedation without restoring slow-wave sleep. This pattern is consistent with the bioelectromagnetic framework: agents that restore the deep-sleep oscillatory dynamics required for neural oscillatory homeostasis tend to relieve pain, while agents that produce sedation without restoring slow-wave architecture do not. Pain relief from sleep aids therefore appears more closely tied to restoration of slow-wave electromagnetic dynamics than to hypnotic action *per se*, though this generalization rests on a relatively small set of well-characterized comparisons and warrants confirmation in larger mechanism-stratified trials (96).

## 6 Hypothesis testing and falsifiability

**Figure 4:**
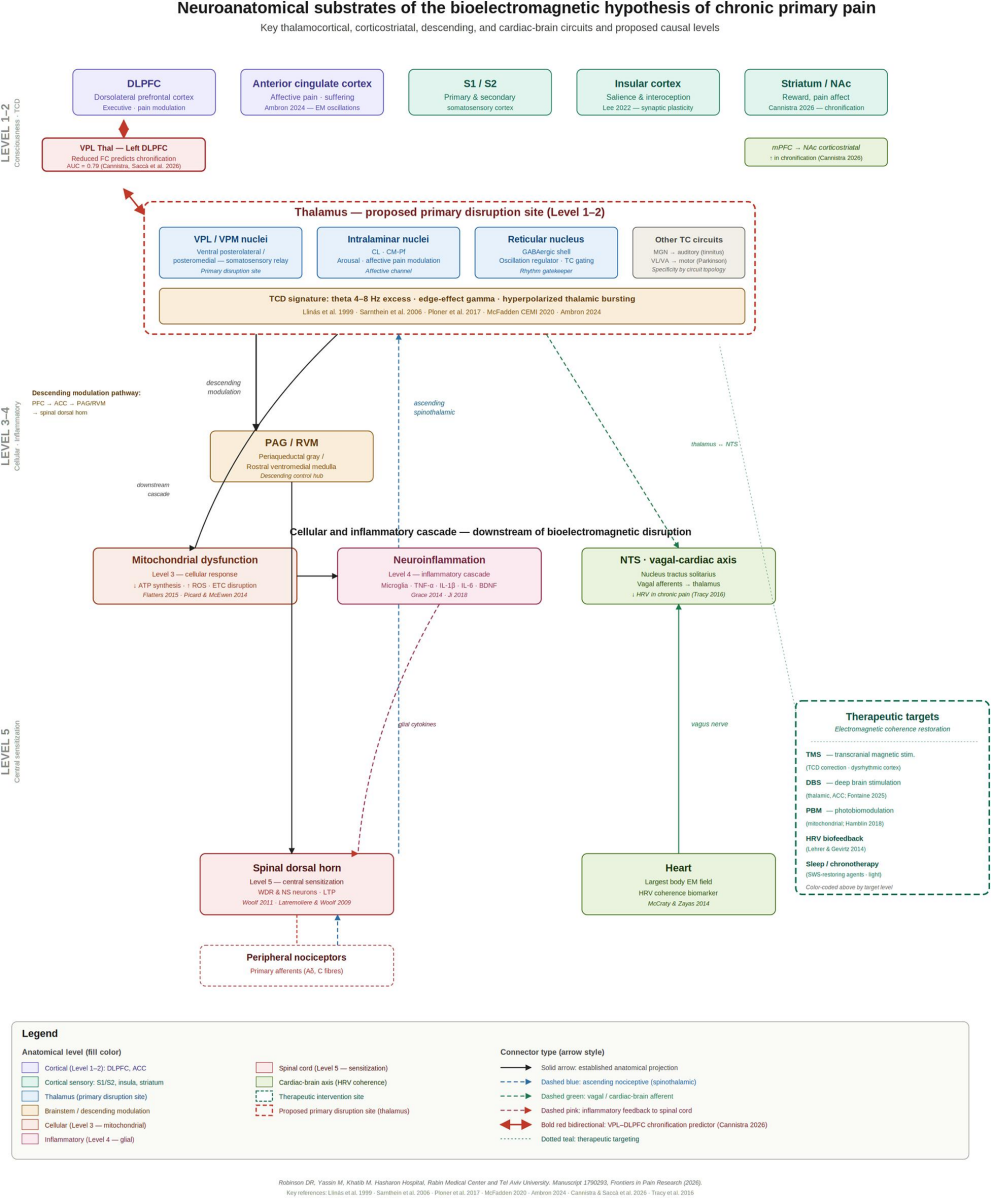
Neuroanatomical substrates of the bioelectromagnetic hypothesis of chronic primary pain. Key thalamocortical, corticostriatal, descending modulatory, and cardiac-brain circuits are shown with their proposed roles in the upstream framework. The thalamus (dashed red border) represents the proposed primary site of bioelectromagnetic disruption (Levels 1–2), with thalamocortical dysrhythmia (TCD) propagating downstream through brainstem, spinal cord, and peripheral circuits. Bold red bidirectional arrows indicate the VPL thalamus–left DLPFC connectivity cluster that prospectively predicts pain chronification with area under the receiver operating curve (AUC) = 0.79 (9). Inset nuclei: VPL/VPM, ventral posterolateral/posteromedial nuclei (somatosensory relay); Intralaminar, centrolateral and centromedian-parafascicular nuclei (affective pain modulation); Reticular nucleus (oscillation gate). Therapeutic targets (dashed green border, right panel) represent the principal electromagnetic intervention sites supported by current evidence. Color coding corresponds to causal levels in Figure 1. ACC, anterior cingulate cortex; DLPFC, dorsolateral prefrontal cortex; DBS, deep brain stimulation; HRV, heart rate variability; NAc, nucleus accumbens; NTS, nucleus tractus solitarius; PAG, periaqueductal gray; PBM, photobiomodulation; RVM, rostral ventromedial medulla; S1/S2, primary/secondary somatosensory cortex; TCD, thalamocortical dysrhythmia; TMS, transcranial magnetic stimulation; VPL/VPM, ventral posterolateral/posteromedial nuclei; WDR, wide dynamic range; NS, nociceptive-specific.

For the hypothesis to be scientific, it must be falsifiable. The following observations would substantially undermine or refute the bioelectromagnetic framework (Table 2):

1. Consistent finding of normal thalamocortical rhythms and HRV coherence in carefully characterized chronic primary pain patients, across multiple well-designed studies.
2. Failure of electromagnetic coherence-restoring interventions (PBM, HRV biofeedback, TMS targeting dysrhythmic regions) to show efficacy in randomized controlled trials despite adequate treatment parameters.
3. Demonstration that central sensitization and neuroinflammation precede (rather than follow) electromagnetic coherence disruption in longitudinal studies of acute-to-chronic pain transition.
4. Complete resolution of chronic pain by purely peripheral interventions without any change in central electromagnetic measures (Figure 4).

**Table 2:** Falsifiability criteria for the bioelectromagnetic hypothesis.

#	Falsifying Observation	Required Study Design	Hypothesis Status if Observed
1	Normal thalamocortical rhythms and HRV coherence in chronic primary pain patients	Well-powered MEG/EEG + HRV studies in fibromyalgia, chronic widespread pain (*n* > 100 each)	Strongly weakened or refuted
2	Failure of EM coherence-restoring interventions (PBM, HRV biofeedback, TMS) vs. sham	Multiple adequately powered RCTs (*n* ≥ 60 per arm; ≥3 independent trials) with optimized treatment parameters	Strongly weakened
3	Central sensitization markers precede electromagnetic coherence disruption	Longitudinal studies (*n* ≥ 150 incident acute pain patients followed ≥6 months) with serial bioelectromagnetic assessments during acute-to-chronic transition	Refuted (causal direction reversed)
4	Complete resolution of chronic pain by peripheral interventions without central EM changes	RCTs (*n* ≥ 80 per arm) of peripheral interventions with concurrent MEG/EEG and clinical outcomes	Refuted (peripheral cause confirmed)

### 6.1 Proposed experiments

#### 6.1.1 Experiment 1 (temporal precedence study)

A prospective cohort study following patients with acute low back pain (*n* = 200) with monthly assessments of MEG-measured thalamocortical oscillations, HRV coherence, and pain intensity over 12 months. Inclusion criteria: acute low back pain onset within 4 weeks, no prior chronic pain history, age 25–65. Primary outcome: whether TCD (defined as theta power >2 SD above healthy controls) and HRV abnormalities (RMSSD <25 ms) precede, accompany, or follow the transition to chronic pain (defined as pain persisting >3 months with NRS ≥4). Secondary outcomes: correlation between degree of electromagnetic abnormality at 1 month and pain status at 12 months; identification of electromagnetic “signatures” predictive of chronification. Based on the (9) finding that VPL-DLPFC thalamocortical connectivity predicts chronification with AUC 0.79, this experiment is powered to detect electromagnetic precedence of chronification if it exists.

#### 6.1.2 Experiment 2 (intervention with biomarkers)

A double-blind randomized controlled trial of photobiomodulation vs. sham in fibromyalgia patients (*n* = 100, 50 per arm). Active treatment: transcranial PBM (810 nm, 250 mW/cm^2^, 20 min, 3x weekly for 8 weeks) targeting prefrontal and motor cortex. Assessments at baseline, 4 weeks, 8 weeks, and 12-week follow-up include: MEG for TCD quantification, HRV analysis (time and frequency domain), fingertip UPE measurement, clinical outcomes (FIQ-R, NRS, BPI). The hypothesis predicts that: (a) clinical improvement should correlate with TCD normalization (*r* > 0.4); (b) responders (≥30% pain reduction) should show greater electromagnetic normalization than non-responders; (c) electromagnetic changes should precede or coincide with clinical improvement, not lag behind it.

#### 6.1.3 Experiment 3 (cross-modal validation)

A cross-sectional study comparing TCD patterns, HRV coherence, UPE emission, and circadian rhythm markers (salivary melatonin, cortisol awakening response) across four matched cohorts (*n* = 50 each): (a) chronic primary pain (fibromyalgia meeting ACR 2016 criteria), (b) chronic secondary pain (knee osteoarthritis with radiographic confirmation), (c) pain-free controls with anxiety disorder, (d) healthy controls. The hypothesis predicts greatest electromagnetic coherence disruption in chronic primary pain, with intermediate disruption in chronic secondary pain.

#### 6.1.4 Experiment 4 (mechanistic cascade study)

A prospective study in patients undergoing planned surgery expected to produce significant postoperative pain (e.g., total knee arthroplasty, *n* = 80). Serial assessments at: pre-operative baseline, postoperative day 3, week 2, week 6, month 3, and month 6. This schedule was deliberately revised from a denser early-postoperative protocol (originally including postoperative days 1 and 7) to reduce participant burden during the acute-recovery window, where repeated MEG and quantitative sensory testing sessions are poorly tolerated and may compromise recruitment and retention. The retained time points still bracket the principal inflammatory and oscillatory transitions of interest: the acute inflammatory peak (days 3–7), the early resolution window (weeks 2–6), and the chronification cut-off at three months. Measures include: MEG (TCD markers), HRV, serum inflammatory markers (CRP, IL-6, TNF-α), central sensitization indicators (temporal summation, conditioned pain modulation), and clinical pain outcomes. The upstream hypothesis predicts that TCD and HRV changes should precede or coincide with inflammatory marker elevation.

#### 6.1.5 Experiment 5 (HRV biofeedback mechanistic trial)

A randomized trial comparing HRV biofeedback training (*n* = 40) vs. relaxation control (*n* = 40) in chronic low back pain. HRV biofeedback group receives 10 sessions of resonance frequency breathing training with home practice. Pre/post assessments include MEG, comprehensive HRV analysis, UPE, and clinical outcomes at baseline, post-treatment, and 3-month follow-up. The hypothesis predicts that improvements in HRV coherence should correlate with TCD normalization and pain reduction.

## 7 Discussion

### 7.1 Strengths of the framework

The bioelectromagnetic field hypothesis offers several potential advantages over purely molecular/cellular models. First, the framework integrates previously disparate observations—TCD, biophoton alterations, HRV abnormalities, circadian disruption, psychological modulation—under a unified theoretical umbrella. Second, by situating the primary pathology at the consciousness-brain interface, the framework directly addresses pain's essential feature as a conscious experience. Third, the frequent failure of peripheral interventions and the efficacy of treatments targeting higher-order processes are expected if the primary disturbance is upstream. Fourth, the framework generates novel predictions and suggests therapeutic approaches targeting electromagnetic coherence restoration. Fifth, the recent prospective neuroimaging data of Cannistra, Saccà et al. (9) and the EM-consciousness-pain work of Ambron (20) provide the first direct empirical anchors for the hypothesis, independent of our own research group, strengthening its evidential basis.

### 7.2 Limitations and caveats

Several important limitations must be acknowledged. While converging evidence is consistent with the hypothesis, direct experimental demonstration of consciousness-brain interface disruption as causal in chronic pain remains to be established. Much of the supporting evidence is correlational or indirect. The transmissive theory of brain function remains outside mainstream neuroscience; the CEMI framework, while more empirically grounded, is also not universally accepted. Chronic pain is heterogeneous, and no single framework may explain all cases—the bioelectromagnetic hypothesis may be more applicable to chronic primary pain than to chronic secondary pain with clear peripheral pathology.

It must also be acknowledged that synaptic plasticity in the pain-related cingulate and insular cortex, as documented by Lee et al. (33), represents a powerful and well-evidenced perpetuating mechanism. Our framework positions this as downstream—a consequence of sustained electromagnetic disruption rather than a primary cause—but direct evidence for this ordering awaits the longitudinal studies proposed above. The framework is intended as a complement to, not a replacement of, established molecular pain mechanisms.

### 7.3 Therapeutic tolerance and the upstream framework

A clinically important observation that our framework addresses is the well-documented phenomenon of diminishing pharmacological efficacy in chronic pain over time. Opioid tolerance, loss of NSAID effect, and the modest long-term outcomes of most pharmacological interventions are not predicted by purely molecular models of central sensitization—once sensitization is established, sustained receptor antagonism or modulation should in principle maintain analgesia. Within the upstream bioelectromagnetic framework, this progressive loss of effect is explicable: pharmacological agents target downstream nodes (receptors, ion channels, cytokines) while leaving the upstream electromagnetic disruption unaddressed. As long as the primary bioelectromagnetic incoherence persists, it continues to drive the downstream molecular cascades, overwhelming the relief provided by downstream targeting. This predicts that the most durable treatments should be those that address the upstream electromagnetic level—consistent with the observation that mind-body practices, HRV biofeedback, and TMS, which more directly target neural oscillatory dynamics, may show more sustained effects than receptor-targeted pharmacotherapy in some patient populations.

### 7.4 Clinical and research implications

The framework suggests several therapeutic directions: (1) Electromagnetic coherence restoration—optimized HRV biofeedback protocols, targeted photobiomodulation, transcranial stimulation targeting TCD; (2) Chronotherapeutic approaches—timed light exposure, melatonin supplementation, sleep optimization aligned with circadian rhythms; (3) Consciousness-level interventions—meditation, contemplative practices, yoga, tai chi may work partly through electromagnetic coherence restoration; (4) Multimodal approaches—interventions targeting multiple levels simultaneously may show synergistic effects; (5) Predictive biomarkers—MEG-measured TCD and HRV coherence at subacute pain presentation may identify patients at risk of chronification, enabling early upstream intervention.

## 5 Conclusions

We have proposed a bioelectromagnetic field hypothesis of chronic pain that situates the primary pathology at the interface where consciousness and neural tissue interact. This framework positions the well-documented mechanisms of chronic pain—central sensitization, neuroinflammation, glial activation, epigenetic modifications—as potential downstream consequences of upstream bioelectromagnetic disruption rather than as primary causes.

The convergence of evidence from optical (UPE), magnetic (MEG/MCG), electrical (EEG/TCD), therapeutic (PBM, HRV biofeedback, DBS, TMS), and prospective neuroimaging domains (9) is consistent with the hypothesis that chronic primary pain may exist as a disruption of the bioelectromagnetic field—upstream of, and causally prior to, the molecular cascades currently targeted by most interventions. The independent theoretical work of McFadden (8) and Ambron (20) on electromagnetic field consciousness and its relationship to conscious pain provides further scaffolding for this framework. As the WHO's recognition of chronic primary pain as a disease demands new conceptual frameworks, the bioelectromagnetic hypothesis offers a scientifically grounded yet paradigm-expanding perspective that may help advance understanding and treatment of one of humanity's most prevalent and disabling conditions.

## Data Availability

Publicly available datasets were analyzed in this study. This data can be found here: De-identified data available upon reasonable request, subject to IRB approval. Requests should be directed to the corresponding author.
